# Improvement in endothelial function in hypertensive patients after Ramadan fasting: effects of cortisol

**DOI:** 10.55730/1300-0144.5603

**Published:** 2023-02-07

**Authors:** Erkan DEMİRCİ, Eyüp ÖZKAN

**Affiliations:** 1 Department of Cardiology, Kayseri City Hospital, Kayseri, Turkey; 2 Department of Cardiology, İstanbul Başakşehir Cam and Sakura City Hospital, İstanbul, Turkey

**Keywords:** Hypertension, Ramadan fasting, endothelial dysfunction, flow-mediated dilatation, cortisol

## Abstract

**Background/aim:**

There are studies on the effects of Ramadan fasting (RF), which is one of the intermittent fasting diets, on both hypertension and endothelial function. However, the relationship between possible improvements in endothelial function and blood pressure after RF is not clear. In this study, we aimed to evaluate the effects of RF on blood pressure and endothelial dysfunction in patients with arterial hypertension (HT).

**Materials and methods:**

Sixty-four HT patients, aged 45–65, who were followed up in the Cardiology Department of Kayseri City Hospital and fasted during Ramadan between 13 April and 13 May 2021 with their self-consents were enrolled to study. Body mass index (BMI), blood pressure, and flow-mediated dilatation (FMD) were assessed before and after Ramadan. Also, 5 mL venous blood samples were taken between 8:00 and 8:30 a.m. from all participants to assess cortisol, C-reactive protein (CRP), and other laboratory data.

**Results:**

In patients, FMD values were found to be higher after Ramadan compared to values before the fasting period (p < 0.001). CRP and cortisol levels decreased after fasting, and the decrease in CRP (95% CI for B = −1.685 – −0.334, p = 0.009) and cortisol levels (95% CI for B = −0.392 – 0.092, p = 0.039) were determined as the predictive factors for FMD after RF.

**Conclusion:**

Endothelial functions as determined by FMD improved after 30 days of intermittent fasting. The decreased CRP and cortisol levels may contribute to the improvement in FMD after RF.

## 1. Introduction

Endothelial dysfunction (ED) is a condition that includes both decreased endothelium-dependent vasodilation and increased endothelial inflammatory activation [1]. Although ED is widely accepted as a predictor of atherosclerosis development and future cardiovascular events, its role in hypertension (HT) is less clear. Nitric oxide (NO) bioavailability appears to play a critical role in the development and progression of HT, diabetes, and atherosclerosis in ED [2]. A large number of studies have found that HT is associated with impaired ED and endothelium-dependent vascular relaxation in the arteries, including the coronary, forearm, and renal arteries, and is also associated with an increased risk of cardiovascular diseases [3–5]. Therefore, enhancement or improvement in endothelial function is expected to prevent the development of atherosclerosis and cardiovascular events. In addition, assessing endothelium-improving action may be beneficial for HT-related cardiovascular events.

ED can be improved by using medications such as statins and acetylsalicylic acid (ASA), as well as aerobic exercise, weight loss, and the Mediterranean diet [6]. Although no studies have been conducted to investigate the effects of RF, particularly on ED in patients with HT, studies have shown that hypertensive patients with combination therapy with RF have a significant improvement in daytime blood pressure (BP) [7,8]. On the other hand, conflicting results were reported. There is a study in the literature in which no change was observed in ambulatory blood pressure after intermittent fasting, and there is another study in which no change was observed in the course of blood pressure after calorie restriction in stage 2–3 hypertensive patients. [9,10].

In our study, considering the different results in the literature and the lack of data on ED; we aimed to evaluate: (i) the effect of Ramadan fasting on BP in hypertensive individuals as well as on clinical data such as lipid profile, weight, and plasma glucose; (ii) possible positive effects of Ramadan fasting on ED; (iii) the relationship between the possible positive effects on ED and the possible change in BP.

## 2. Methods

### 2.1. Study population

Our study is a prospective cohort study. The study population consists of 64 patients, aged from 45 to 65, with a diagnosis of HT who were followed up on in Kayseri City Hospital’s Cardiology Department between February 10 and May 17, 2021, and fasted for the entire Ramadan (from April 13 to May 13) with their consent. Patients with essential HT above the second grade, secondary HT, prior coronary artery disease, kidney or liver dysfunction, diabetes, a body mass index (BMI) > 30 kg/m^2^, and any chronic inflammatory disease were excluded. Also, patients who did not comply with the diet for more than 2 days for any reason (including menstrual bleeding) were not included in the study. To limit calorie intake variability, the patients were asked to follow a 16-h intermittent fasting routine, with no more than one major meal after sunset and one lighter meal before sunrise. Patients who were evaluated 3 days before Ramadan were assessed for the second time 3 days after Ramadan. BP, heart rate, weight, and laboratory data such as plasma glucose, and lipid profile were assessed twice, as well as flow-mediated dilatation (FMD) before and after Ramadan.

From all participants, 5 mL venous blood samples were collected between 08:00 and 08:30 a.m. Routine biochemical parameters were analyzed on Cobas 8000 modular autoanalyzer (C702, Roche Diagnostics, Mannheim, Germany) using manufacturer’s kits (by spectrophotometric/enzymatic methods). Cortisol was measured on Elecsys (E602, Roche Diagnostics, Mannheim, Germany) immunoassay automated analyzer using a manufacturer’s kit (Elecsys Cortisol II assay) by competitive immunoassay method.

Before participating in the study, all participants were given signed informed consent. The Kayseri City Hospital’s ethics committee (04.02.2021/284) approved the project.

### 2.2. Assessment of flow-mediated dilatation

Due to its easy approach and noninvasive nature, FMD measured by Doppler ultrasound has emerged as the most preferred clinical research tool for assessing vascular endothelial function [11]. In our study, FMD, a surrogate marker of endothelial function, was measured from the brachial artery. According to reports, proper subject preparation is critical for a successful ultrasonographic assessment of FMD. As a result, rigorous compliance to vitamin supplementation restrictions, medication discontinuance (or at least documentation), caffeine usage, menstrual cycle phase, physical activity, and being fasted and resting before the FMD test are critical [12]. The pharmacotherapy of patients was limited with angiotensin-converting enzyme inhibitors (ACEi) and angiotensin-receptor blockers (ARB) considering the effects of drugs such as beta-blockers, nitrates, and calcium channel blockers, etc., on FMD [11,12]; therefore, patients who received medications other than ACEi and ARB were excluded from the study. The assessments were made in accordance with the recommendations in the literature [11,12]. An ultrasound system with high resolution (Philips EPIQ 7G, USA) was used. Using a 7.5-MHz linear array transducer, the diameter of the brachial artery was calculated from two-dimensional ultrasound pictures (FMD: (reactive hyperemia diameter baseline diameter)/baseline diameters 100%). Participants were asked to lie down for at least 5 min in a calm, 23 °C temperature-controlled room to get their resting BP using a standard sphygmomanometer on their left arm. At first, the brachial artery’s baseline diameter was determined. The occlusion cuff was fixed at 50 mmHg higher than systolic blood pressure (SBP) for 5 min. After the forearm had been ischemic for five minutes, the cuff was turned off, and the brachial artery was observed for two minutes.

### 2.3. Statistical analysis

The data were analyzed using IBM SPSS Statistics 21.0 (IBM North America, New York, USA). Summary statistics of the data were given several units (n), percent (%), mean, standard deviation, median, minimum, and maximum values. The distribution of the data was analyzed using the Shapiro-Wilk test and Q-Q plot normality. To compare variables, a paired-samples-test was used. Partial correlation analysis was used to assess the correlation of parameters when controlling the effects of age and sex, and the predictive effects of some parameters on FMD (%) were evaluated using the linear regression analysis method. A p-value smaller than 0.05 was accepted as statistically significant.

## 3. Results

The baseline characteristics of the participants were given in Table 1. Weight and BMI decreased after Ramadan fasting, but changes were not statistically significant (p = 0.073, p > 0.05), as well as fasting glucose (p = 0.143) (Table 2). Compared to levels that were measured before Ramadan, patients had lower LDL (p = 0.034), CRP (p < 0.001), serum cortisol (p = 0.044), and SBP and diastolic BP (DBP) levels (p = 0.032, p = 0.043) after fasting (Table 2). In contrast, FMD values increased after Ramadan fasting (p < 0.001) (Table 2).

In the correlation analysis, we found that there was a negative correlation between FMD and SBP (r = −0.294, p = 0.032). Furthermore, a negative correlation was found between CRP and FMD as well as cortisol and FMD (r = −0.659, p = 0.023; r = −0.271, p = 0.031) (Table 3). Also, LDL was negatively correlated with FMD (r = −0.725, p = 0.022) (Table 3).

In the linear regression analysis (R square = 0.702; p = 0.004), the decrease in CRP (95% CI for B = −1.685 − (−0.334), p = 0.009) and cortisol levels (95% CI for B = −0.392 – 0.092, p = 0.039) were determined as the predictive factors for FMD after RF as well as a decrease in SBP (95% CI for B = −3.353 – 0.574, p = 0.031) and a decrease in LDL (95% CI for B = −0.705 – 0.034, p = 0.039) (Table 4).

## 4. Discussion

In the present study, we found that RF, which is a form of intermittent fasting, improved metabolic parameters including LDL, CRP, and cortisol levels, and decreased systolic and diastolic BP. More importantly, patients had better endothelial functions evaluated by FMD, after fasting.

It is widely recognized that HT is one of the most frequent disorders among the elderly and that the prevalence of HT rises significantly with age. HT accelerates aging-related changes in vascular structure and function, especially endothelial function [13]. Studies have found that HT is linked to endothelium-dependent vascular relaxation impairment and ED, which are linked to the development of atherosclerosis and cardiovascular diseases [1,13–15]. Therefore, it can be predicted that interventions that will have a positive effect on HT may also have curative effects on impaired endothelial function.

Increasing evidence points to the potential benefits of RF on BP and endothelial function. In one study, fourteen healthy males fasting during the Ramadan period were assessed before fasting, one month after fasting, and thirty days thereafter [16]. It was found that intermittent fasting improved endothelial-nonendothelial-dependent vasodilation and BP [16]. Another study demonstrated that fasting therapy positively affects endothelial function and reduces arterial injury markers in overweight and obese individuals [17]. Also, it was reported that RF reduces cardiac stress in patients with controlled HT without affecting their hypertensive state [18]. However, there is no human study on the effects of Ramadan fasting on ED in hypertensive patients. In our study, we found that systolic and diastolic BPs were lower in the patients after Ramadan fasting and that Ramadan fasting improved FMD in patients with controlled HT. Again, the decrease in SBP was a predictive factor of the improvement in FMD (%). LDL cholesterol levels were found to be lower after fasting. In addition, fasting glucose, weight, BMI, and triglyceride levels decreased while HDL levels increased in the patients after RF, but the changes were not statistically significant. In studies, it was reported that RF could affect fasting glucose, weight, and BMI positively in special groups including diabetes and HT, and it was suggested that randomized controlled trials focus on long-term clinical outcomes [19,20]. Also, it was reported that a significant increase in HDL and a decrease in TG and LDL in patients with HT [21], and a significant increase in HDL, with a decrease in LDL, was seen after Ramadan in healthy individuals [22]. However, there is no study evaluating the relationship between LDL levels and the improvement of both FMD and BP in hypertensive patients. There is a negative correlation between LDL and FMD but no correlation was found with BP. Also, we found that lower LDL levels could be a predictor of improvement in FMD.

Studies have reported that intermittent fasting reduces oxidative stress [23,24]. Yousefi et al. found that nitric oxide (NO) levels increase after RF compared with the baseline in male patients with cardiovascular risks [25]. HT, on the other hand, is known to cause ED due to decreased NO levels [3,26]. On this point, a dysfunctioning endothelium may lose its ability to protect the vascular system by reducing antithrombotic and/or antiatherosclerotic actions [3,26]. In addition, like NO, CRP and ferritin are also inflammatory biomarkers, and studies showed that fasting was associated with a significant decrease in CRP levels [27,28]. Prolonged intermittent fasting, such as Ramadan, has some beneficial effects on inflammatory markers like homocysteine and CRP [27, 28]. In our study, a decrease in CRP levels was observed after RF. Again, CRP was negatively correlated with FMD (%), and a decreasing CRP level was a predictive factor of the improvement in FMD (%).

It is well known that cortisol levels change during fasting, and changes could differ depending on measurement time [29,30]. In one study, in women with polycystic ovarian syndrome, serum cortisol levels decreased after Ramadan compared to the beginning of Ramadan [31]. In another study, the baseline cortisol level was significantly higher at 08:00 hours than at 20:00 hours, and the cortisol response was significantly lower during Ramadan compared to the time before Ramadan in healthy people [32]. In a recent study with thirty-four healthy individuals, venous blood samples were collected five times: a week before RF, the middle of RF, the last days of RF, one week after RF, and one month after RF. Cortisol levels were found to be significantly lower on the final days of RF and one week after RF compared to one week before RF [33]. Furthermore, in contrast to Al Rawi et al. [34], Bahijri et al. [35] reported a decrease in cortisol levels after RF. However, there is a lack of information on how cortisol is altered in HT and how cortisol affects FMD during RF. In our study, basal cortisol levels were found to be lower after fasting compared to before RF in HT patients. In addition, cortisol level was negatively correlated with FMD, and in a regression analysis, a decrease in cortisol level was assessed as a predictive factor for the improvement in FMD after RF.

There are some limitations of our study. The number of participants was small, Participants consisted only of patients with low cardiovascular risk. Other limitations are that the duration was limited to 1 month and patients with impaired glucose tolerance were not excluded. Future comprehensive studies with longer duration are needed to determine whether IF applications are effective in patients with higher cardiovascular risk (such as diabetes, coronary artery disease, chronic kidney failure, and cerebrovascular disease).

In conclusion, our findings suggest that Ramadan fasting, a form of intermittent fasting has favorable effects on CRP, LDL, and serum cortisol levels, which may contribute to improving endothelial functions determined by FMD. Our findings are hypothesis-generating and for identifying causal relationships, large randomized clinical trials are needed.

## Figures and Tables

**Table 1:** The characteristics of the participants.

Patients with HT	N = 64

Age (years)	50.8 ± 9.9

Sex	28 female/36 male

BMI (kg/m^2^)	25.1 ± 4.2

Duration of HT (years)	4.5 ± 3.05

Drugs used	
ACEi	39 (60.93 %)
ARB	25 (39.06 %)

Hypercholesterolemia	9 (14.06 %)

BMI: body mass index, HT: hypertension, ACEi: angiotensin-converting enzyme inhibitors, ARB: angiotensin-receptor blockers.

**Table 2:** Comparison of clinical parameters in patients with HT before and after Ramadan fasting.

	Before Ramadan fasting	After Ramadan fasting	p-values
Weight (kg)	79.5 ± 12.8	78.5 ± 11.7	p = 0.073
BMI (kg/m^2^)	25.1 ± 4.2	24.6 ± 3.9	p > 0.05
FMD (%)	8.7 ± 3.8	10.8 ± 3.7	**p < 0.001**
HDL (mg/dL)	44.3 ± 10.5	45.2 ± 10.6	p = 0.074
LDL (mg/dL)	142.5 ± 28.8	131.3 ± 27.7	**p = 0.034**
Triglyceride (mg/dL)	216.1 ± 123.0	210.2 ± 103.0	p = 0.056
Fasting glucose (mg/dL)	118.28 ± 30.05	115.53 ± 26.87	p = 0.146
Na (mmol/L)	135.6 ± 1.0	137.1 ± 1.2	**p < 0.05**
Ca (mg/dL)	9.36 ± 0.24	9.05 ± 0.21	**p < 0.05**
Hgb (g/dL)	15.08 ± 1.75	14.94 ± 1.76	p = 0.226
CRP (mg/L)	4.58 ± 2.9	3.29 ± 2.09	**p < 0.001**
Cortisol (μg/dL)	25.25 ± 15.75	22.43 ± 11.45	**p = 0.044**
Systolic blood pressure (mm/Hg)	136.30 ± 15.45	128.43 ± 10.51	**p = 0.032**
Diastolic blood pressure (mm/Hg)	82.78 ± 8.26	77.36 ± 6.64	**p = 0.043**

BMI: body mass index, FMD: flow-mediated dilatation, HDL: high-density lipoprotein, LDL: low-density lipoprotein, Na: sodium, Ca: calcium, Hg: hemoglobin, CRP: C-reactive protein.

**Table 3:** Correlation between changes in FMD and clinical parameters after Ramadan fasting.

	FMD (After fasting)	Systolic blood pressure (After fasting)	Diastolic blood pressure (After fasting)
BMI	r = 0.063 p = 0.607	r = 0.124 p = 0.271	r = 0.053 p = 0.660
LDL	**r =** −0.725* p = 0.022	r = −0.178 p = 0.121	r = −0.153 p = 0.185
HDL	r = 0.091 p = 0.417	r = 0.053 p = 0.646	r = −0.110 p = 0.340
Triglyceride	r = −0.106 p = 0.108	r = −0.122 p = 0.292	r = 0.066 p = 0.570
Fasting glucose	r = 0.167 p = 0.453	r = 0.053 p = 0.722	r = 0.136 p = 0.427
Na	r = 0.043 p = 0.780	r = 0.137 p = 0.274	r = 0.123 p = 0.567
Ca	r = 0.098 p = 0.423	r = 0.128 p = 0.247	r = 0.022 p = 0.934
CRP	**r =** −0.659* p = 0.023	r = −0.047 p = 0.786	r = 0.102 p = 0.350
Cortisol	**r =** −0.271* p = 0.031	r = 0.086 p = 0.509	r = 0.197 p = 0.141

** p < 0.001;

* p < 0.050.

Partial correlation test. r=correlation coefficient. Control variables; age and sex.

**Table 4:** Predictive Factors of FMD after fasting with linear regression model.

Variables	B	SE	Beta	95% CI for B	Sig
Age	−0.110	0.067	−0.027	−0.245 – 0.025	0.108
Weight	−0.058	0.203	0.171	−0.248 – 0.716	0.568
BMI	−0.047	0.234	0.149	−0.342 – 0.678	0.675
Systolic blood pressure	−2.423	0.466	−0.097	−3.353 – 0.574	**0.031**
Diastolic blood pressure	−0.169	0.603	−0.002	−1.005 – 1.234	0.211
LDL	−0.369	0.168	−0.065	−0.705 – 0.034	**0.039**
HDL	0.015	0.012	0.030	−0.010 – 0.039	0.237
Triglyceride	−0.183	0.057	−0.097	−0.235 – 0.168	0.103
Fasting glucose	0.049	0.027	0.022	−0.015 – 0.103	0.168
CRP	−1.009	0.339	−0.890	−1.685 – (−0.334)	**0.009**
Cortisol	−0.204	0.188	−0.287	−0.392 – 0.092	**0.039**

SE: standard error, CI: confidence interval. Enter method (R^2^ = %70.2, Durbin-Watson = 1.847, Cook’s distance = 0.00–0.054).
